# Prediction model of obstructive sleep apnea–related hypertension: Machine learning–based development and interpretation study

**DOI:** 10.3389/fcvm.2022.1042996

**Published:** 2022-12-05

**Authors:** Yewen Shi, Lina Ma, Xi Chen, Wenle Li, Yani Feng, Yitong Zhang, Zine Cao, Yuqi Yuan, Yushan Xie, Haiqin Liu, Libo Yin, Changying Zhao, Shinan Wu, Xiaoyong Ren

**Affiliations:** ^1^Department of Otorhinolaryngology Head and Neck Surgery, The Second Affiliated Hospital of Xi’an Jiaotong University, Xi’an, China; ^2^Molecular Imaging and Translational Medicine Research Center, State Key Laboratory of Molecular Vaccinology and Molecular Diagnostics, Xiamen University, Xiamen, China; ^3^Department of Otorhinolaryngology Head and Neck Surgery, Xi’an Central Hospital, Xi’an, China; ^4^Eye Institute of Xiamen University, School of Medicine, Xiamen University, Xiamen, China

**Keywords:** obstructive sleep apnea, hypertension, machine learning, risk factor, Shapley additive explanations, gradient boosting machine (GBM)

## Abstract

**Background:**

Obstructive sleep apnea (OSA) is a globally prevalent disease closely associated with hypertension. To date, no predictive model for OSA-related hypertension has been established. We aimed to use machine learning (ML) to construct a model to analyze risk factors and predict OSA-related hypertension.

**Materials and methods:**

We retrospectively collected the clinical data of OSA patients diagnosed by polysomnography from October 2019 to December 2021 and randomly divided them into training and validation sets. A total of 1,493 OSA patients with 27 variables were included. Independent risk factors for the risk of OSA-related hypertension were screened by the multifactorial logistic regression models. Six ML algorithms, including the logistic regression (LR), the gradient boosting machine (GBM), the extreme gradient boosting (XGBoost), adaptive boosting (AdaBoost), bootstrapped aggregating (Bagging), and the multilayer perceptron (MLP), were used to develop the model on the training set. The validation set was used to tune the model hyperparameters to determine the final prediction model. We compared the accuracy and discrimination of the models to identify the best machine learning algorithm for predicting OSA-related hypertension. In addition, a web-based tool was developed to promote its clinical application. We used permutation importance and Shapley additive explanations (SHAP) to determine the importance of the selected features and interpret the ML models.

**Results:**

A total of 18 variables were selected for the models. The GBM model achieved the most extraordinary discriminatory ability (area under the receiver operating characteristic curve = 0.873, accuracy = 0.885, sensitivity = 0.713), and on the basis of this model, an online tool was built to help clinicians optimize OSA-related hypertension patient diagnosis. Finally, age, family history of hypertension, minimum arterial oxygen saturation, body mass index, and percentage of time of SaO_2_ < 90% were revealed by the SHAP method as the top five critical variables contributing to the diagnosis of OSA-related hypertension.

**Conclusion:**

We established a risk prediction model for OSA-related hypertension patients using the ML method and demonstrated that among the six ML models, the gradient boosting machine model performs best. This prediction model could help to identify high-risk OSA-related hypertension patients, provide early and individualized diagnoses and treatment plans, protect patients from the serious consequences of OSA-related hypertension, and minimize the burden on society.

## Introduction

Obstructive sleep apnea (OSA) is a sleep disorder characterized by intermittent hypoxemia, autonomic fluctuation, and sleep fragmentation. As of 2019, the prevalence of OSA aged 30–69 years (men and women) in China has reached 24.2%, ranking first in the world (1). Aside from the fact that OSA causes difficult symptoms, many studies demonstrated that OSA is closely associated with many complications, such as cardiovascular diseases, metabolic disorders, and cognitive impairment (2–4). Among them, cardiovascular diseases have received extensive attention because of their serious consequences and high morbidity, especially hypertension. Observational studies have illustrated that 45–68% of subjects with OSA have hypertension (5, 6), and the prevalence of OSA is more than 30% among hypertension patients (7).

Hypertension that is primarily caused or exacerbated by OSA is called OSA-related hypertension after excluding other definite secondary etiologies (e.g., renal artery stenosis, renal parenchymal disease, primary aldosteronism, pheochromocytoma, and Cushing’s disease) (8). In addition, OSA-related hypertension is characterized by high rates of masked hypertension, elevated nocturnal blood pressure, a non-dipper pattern of nocturnal hypertension, and an increased blood pressure variability (9). Notably, patients with OSA and hypertension seem to be associated with more severe outcomes. Studies based on ambulatory blood pressure monitoring (ABPM) showed that participants with a non-dipper pattern of nocturnal hypertension and those who have elevated blood pressure at night demonstrate a greater degree of end-organ damage, higher risk of stroke, increased risk of incident heart failure, and increased risk of renal disease progression (10). Regrettably, OSA-related hypertension is easily disregarded by patients.

As for the general population, the reference method for blood pressure testing is primarily an in-office measurement. However, this diagnostic method is unreliable in the OSA population because of the specific characteristics of OSA-related hypertension. Previous studies have shown that among OSA patients, masked hypertension was found in 30% of patients, and white-coat hypertension was found in approximately 33% of patients (11–13). It means that there is a high risk that OSA patients may be underdiagnosed or overdiagnosed with hypertension. The application of ABPM to systematically and correctly assess blood pressure is recommended in clinically normotensive OSA patients (14). However, ABPM is not cost-efficient and often burdensome, and in clinical practice, it seems challenging to propose ABPM to all OSA patients with normal clinic blood pressure. Thus, the necessity of a simple and convenient clinical tool to assess OSA-related hypertension in daily clinical practice is emphasized, which can allow the use of ABPM selectively rather than routinely.

Machine learning (ML) has been widely developed and used in the medical field because of its remarkable performance in recent years. It can extract information from complex and non-linear data, establish models through science, reveal hidden dependencies between factors and diseases in the big data environment, and help clinicians better understand the diseases (15). Especially in cardiovascular diseases, machine learning has a wide range of applications and satisfactory diagnostic performance. For example, Ward et al. demonstrated that the gradient boosting machine (GBM) model has good discrimination for atherosclerotic cardiovascular disease risk (16). Although ML has gained extensive attention because of its powerful predictive capabilities, it is often criticized for being a black box model, making it hard for clinicians to understand and trust these complex models. Hence, this has limited its widespread use in medical decision-making (17).

Timely blood pressure screening and early accurate identification of OSA-related hypertension are crucial in minimizing the associated negative health effects. Regrettably, no ML models are available to predict the risk of OSA-related hypertension. In this study, we aimed to develop ML-based prediction models for OSA-related hypertension based on available clinical data from patients to identify high-risk patients. In addition, we used Shapley additive explanations (SHAP) (18), a method for interpreting results made by machine learning models, to explore the relationship between features and the risk of OSA-related hypertension. In addition, we further provide individual interpretations of the model’s decisions through SHAP. Moreover, we established a web-based risk calculator based on the most predictive maximum likelihood algorithm to promote its clinical application, which provided clinicians with valuable tools for risk assessment in OSA-related hypertension.

## Materials and methods

### Study design and subjects

This is a retrospective observational study. It retrospectively included the OSA patients admitted to the Department of Otorhinolaryngology—Head and Neck Surgery of the Second Affiliated Hospital of Xi’an Jiaotong University between October 2019 and December 2021. All study subjects underwent nighttime polysomnography or home sleep apnea testing and blood pressure monitoring, additionally, cardiologists assessed their blood pressure. OSA was diagnosed on the basis of apnea–hypopnea index (AHI) ≥ 5 events per hour through polysomnography (19). Hypertension was defined as a previous diagnosis with current antihypertensive therapy. Additionally, patients with elevated nocturnal blood pressure who had no history of hypertension were further examined and identified as newly diagnosed with hypertension by a cardiologist with more than 10 years of working experience. The definition of hypertension is described in detail in the Supplementary material.

The inclusion criteria were as follows: (1) patients with age ≥ 18 years, (2) patients with AHI ≥ 5 events per hour, and (3) patients who have not received OSA-related treatment in the past. The exclusion criteria were as follows: (1) patients with incomplete baseline data; (2) patients with disease potentially affecting blood pressure regulation, such as multiple organ dysfunction syndrome, uremia, severe cardiac heart failure, renal, or cardiac transplantation; (3) patients with the most common causes of secondary hypertension, namely, renal parenchymal disease, renovascular diseases, coarctation of the aorta, Cushing’s syndrome, primary hyperaldosteronism, pheochromocytoma, hyperthyroidism, and hyperparathyroidism; (4) pregnant women; (5) patients with history of snoring shorter than the duration of hypertension; and (6) patients who were diagnosed with central sleep apnea (central AHI ≥ 5 events per hour).

This study was approved by the ethics committee of the Second Affiliated Hospital of Xi’an Jiaotong University (approval no. 2021031). In addition, all patients who participated in the research provided informed consent. The inspection items and processes involved in this study are in line with the Declaration of Helsinki.

### Data elements

Twenty-seven relevant clinical indicators were collected, and overall, the 27 candidate variables included were as follows: (1) demographic characteristics, namely, gender, heart disease family history of hypertension, diabetes, hypothyroidism, body mass index (BMI), waist circumference, neck circumference, and age/10; (2) lifestyle behaviors, namely, drinking, smoking, high-salt diet, high-fat diet, poor sleep quality, sedentariness, emotionally stable, mental stress, and smoking amount; and (3) OSA-related medical history and indicators, namely, memory decline, inattention, Epworth Sleepiness Scale (20), course of snoring, course of choking, AHI, obstructive apnea index (OAI), minimum arterial oxygen saturation/10 (minimum SaO_2_/10), and percentage of time of SaO_2_ < 90%/10 (CT90/10).

### Development and validation of prediction models

By comparing the clinical characteristics of the hypertension and non-hypertension groups, the risk factors for predicting OSA-related hypertension were analyzed using the univariate analysis, and they were incorporated into machine learning as characteristic variables. Additionally, they were also used in the multivariate logistic regression analysis to obtain independent predictors associated with OSA-related hypertension.

All patients were randomly divided into a training set for constructing the predictive model and a test set for the model validation at a ratio of 7:3. The following six representative supervised ML algorithms were used for model construction in the training dataset: adaptive boosting, GBM, multilayer perceptron, bootstrapped aggregating, logistic regression, and extreme gradient boost (21–24). During training, the training cohort internal validation method used 10-fold cross-validation to evaluate the predictive power of each ML classifier in plotting the average area under the receiver operating characteristic curve (AUC). With the use of the validation cohort, the receiver operating characteristics of the six ML models were plotted, and AUC values were calculated to evaluate the predictive ability of the different models in cohorts. By comparing the predictive performance of our ML models, the model with the best predictive performance was selected as the final model. In addition, a confusion matrix was used to evaluate the prediction model performance. Subsequently, on the basis of the best predictive ability model, an online risk calculator that can make predictions using newly entered data of OSA patients was created.

### Model interpretation

Shapley additive explanations (SHAP) is a model-agnostic explanation technique based on cooperative game theory that helps interpret the results from a predictive model. The interpretation is based on quantifying the SHAP value for each feature, representing the contribution of a feature to the predicted risk of OSA-related hypertension (25, 26). For each sample, the model produces a prediction value, and the sum or average of the absolute Shapley value of each feature of all individuals is the overall feature importance. Components with large fundamental Shapley values are very important. In addition, the SHAP method also proves each feature value’s positive or negative influence on the predicted results, similar to coefficient values in logistic regression. A positive SHAP value indicates that the corresponding feature contributes to a higher risk of the result, whereas a negative SHAP value indicates that the corresponding feature leads to a lower risk of the result. To determine the main predictors of OSA-related hypertension, we identified the importance of ranking features from the final model through the SHAP summary plot and provided individual interpretations of the model’s decisions.

### Statistical analysis

All analyses and random division of training and validation sets were performed with R software (version 3.6.0). Continuous variables were represented as the median (p25, p75), whereas categorical variables were represented as numbers (n) and proportions (%). The Wilcoxon rank-sum test compared the two groups’ differences for continuous variables, and categorical variables were evaluated using the chi-squared test. Logistic regression analysis was used to analyze the relationship between various predictor variables (either categorical or continuous) and an outcome that is binary (dichotomous). The Python programming language (version 3.8) was also used to develop and evaluate ML models and design network calculators. For model interpretation, the SHAP was implemented using the Python Shap package. *P* < 0.05 was considered statistically significant.

## Results

### Patient characteristics

**FIGURE 1:**
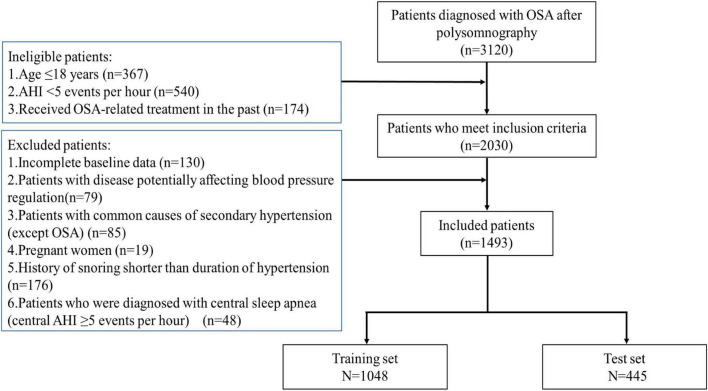
Summary of patient inclusion. AHI, apnea–hypopnea index; OSA, obstructive sleep apnea.

After the screening process, a total of 1,493 OSA patients were eligible for the study (Figure 1). The baseline characteristics of these patients are summarized in Table 1. For the demographic variables, the two groups were significantly different in heart disease, family history of hypertension, diabetes, BMI, waist circumference, neck circumference, and age/10 (all *P* < 0.05). For the lifestyle behavior variables, high-salt diet, poor sleep quality, and smoking amount were significant variables (all *P* < 0.05). For OSA-related medical history and indicators, memory decline, Epworth Sleepiness Scale, course of snoring, course of choking, AHI, OAI, minimum SaO_2_/10, and CT90/10 were all significantly different between the two groups (all *P* < 0.05).

**TABLE 1:** Demographic and clinical characteristics.

Characteristic	Total (*n* = 1,493)	Non-hypertension (*n* = 1,134)	Hypertension (*n* = 359)	*P* value
**Gender, n (%)**				0.143
Female	224 (15.0)	161 (14.2)	63 (17.5)	
Male	1,269 (85.0)	973 (85.8)	296 (82.5)	
**Heart disease, n (%)**				< 0.001
No	1,394 (93.4)	1,085 (95.7)	309 (86.1)	
Yes	99 (6.6)	49 (4.3)	50 (13.9)	
**Family history of hypertension, n (%)**				< 0.001
No	1,063 (71.2)	905 (79.8)	158 (44.0)	
Yes	430 (28.8)	229 (20.2)	201 (56.0)	
**Diabetes, n (%)**				< 0.001
No	1,437 (96.2)	1,113 (98.1)	324 (90.3)	
Yes	56 (3.8)	21 (1.9)	35 (9.7)	
**Hypothyroidism, n (%)**				0.278
No	1,464 (98.1)	1,109 (97.8)	355 (98.9)	
Yes	29 (1.9)	25 (2.2)	4 (1.1)	
Body mass index, median (Q1, Q3)	26.7 (24.6, 29.4)	26.2 (24.4, 28.7)	28.1 (25.9, 30.5)	< 0.001
Waist circumference, median (Q1, Q3)	98.0 (92.0, 105.0)	97.0 (92.0, 103.0)	102.0 (95.0, 108.0)	< 0.001
Neck circumference, median (Q1, Q3)	40.0 (37.5, 42.0)	39.0 (37.0, 41.0)	41.0 (38.0, 43.0)	< 0.001
Age/10, median (Q1, Q3)	4.0 (3.3, 4.9)	3.7 (3.2, 4.6)	4.8 (4.0, 5.6)	< 0.001
**Drinking, n (%)**				0.965
No	901 (60.3)	684 (60.3)	217 (60.4)	
Yes	592 (39.7)	450 (39.7)	142 (39.6)	
**Smoking, n (%)**				0.112
No	834 (55.9)	647 (57.1)	187 (52.1)	
Yes	659 (44.1)	487 (42.9)	172 (47.9)	
**High-salt diet, n (%)**				< 0.05
No	1,124 (75.3)	869 (76.6)	255 (71.0)	
Yes	369 (24.7)	265 (23.4)	104 (29.0)	
**High-fat diet, n (%)**				0.069
No	1,049 (70.3)	811 (71.5)	238 (66.3)	
Yes	444 (29.7)	323 (28.5)	121 (33.7)	
**Poor sleep quality, n (%)**				< 0.05
No	808 (54.1)	634 (55.9)	174 (48.5)	
Yes	685 (45.9)	500 (44.1)	185 (51.5)	
**Sedentariness, n (%)**				0.182
No	573 (38.4)	424 (37.4)	149 (41.5)	
Yes	920 (61.6)	710 (62.6)	210 (58.5)	
**Emotionally stable, n (%)**				0.157
No	416 (27.9%)	305 (26.9)	111 (30.9)	
Yes	1,077 (72.1%)	829 (73.1)	248 (69.1)	
**Mental stress, n (%)**				0.099
No	1,092 (73.1)	842 (74.3)	250 (69.6)	
Yes	401 (26.9)	292 (25.7)	109 (30.4)	
Smoking amount, median (Q1, Q3)	0.0 (0.0, 1.0)	0.0 (0.0, 1.0)	0.0 (0.0, 1.0)	< 0.05
**Memory decline, n (%)**				< 0.01
No	549 (36.8)	439 (38.7)	110 (30.6)	
Yes	944 (63.2)	695 (61.3)	249 (69.4)	
**Inattention, n (%)**				0.744
No	687 (46.0)	525 (46.3)	162 (45.1)	
Yes	806 (54.0)	609 (53.7)	197 (54.9)	
Epworth sleepiness scale, median (Q1, Q3)	9 (6, 14)	8.5 (5, 13)	11 (7, 16)	< 0.001
Course of snoring, median (Q1, Q3)	9.0 (4.0, 12.0)	8.0 (3.6, 10.0)	10.0 (6.0, 20.0)	< 0.001
Course of choking, median (Q1, Q3)	3.0 (1.0, 6.0)	2.0 (1.0, 5.0)	4.0 (1.0, 8.0)	< 0.001
AHI, median (Q1, Q3)	46.2 (23.0, 67.9)	41.8 (19.8, 66.1)	57.7 (33.1, 70.8)	< 0.001
OAI, median (Q1, Q3)	21.1 (5.2, 46.6)	17.5 (4.2, 43.5)	32.5 (14.7, 53.1)	< 0.001
Minimum SaO_2_/10, median (Q1, Q3)	7.6 (6.5, 8.4)	7.9 (6.8, 8.5)	6.9 (5.7, 7.8)	< 0.001
CT90/10, median (Q1, Q3)	0.7 (0.1, 3.0)	0.5 (0.1, 2.6)	1.5 (0.3, 4.0)	< 0.001

AHI, apnea–hypopnea index; OAI, obstructive apnea index; SaO_2_, arterial oxygen saturation; CT90/10, percentage of time of SaO_2_ < 90%/10.

### Univariate and multivariate logistic regression

Variables with a *P* < 0.05 in the univariate analysis were selected for multivariate logistic regression analysis to identify the independent risk factors of OSA-related hypertension patients (Table 2), and all regression coefficients are shown in Supplementary Table 1. In addition, the results indicated that family history of hypertension, BMI, age/10, minimum SaO_2_/10, and CT90/10 were independent risk factors for OSA-related hypertension (all *P* < 0.05).

**TABLE 2:** Univariate analysis and multivariate logistic regression analysis of variables.

		Univariate analysis		Multivariate analysis	
Characteristics	Category	OR (95% CI)	*P* value	OR (95% CI)	*P* value
Gender	Female	Ref	Ref	Ref	Ref
	Male	0.777 (0.565–1.070)	0.122	–	–
Heart disease	No	Ref	Ref	Ref	Ref
	Yes	3.583 (2.369–5.419)	< 0.001	1.437 (0.845–2.446)	0.181
Family history of hypertension	No	Ref	Ref	Ref	Ref
	Yes	5.027 (3.9–6.48)	< 0.001	5.388 (3.975–7.302)	< 0.001
Diabetes	No	Ref	Ref	Ref	Ref
	Yes	5.725 (3.287–9.973)	< 0.001	1.849 (0.934–3.662)	0.078
Hypothyroidism	No	Ref	Ref	Ref	Ref
	Yes	0.500 (0.173–1.446)	0.201	–	–
Body mass index	–	1.148 (1.109–1.188)	< 0.001	1.121 (1.038–1.210)	< 0.01
Waist circumference	–	1.051 (1.039–1.064)	< 0.001	1.014 (0.984–1.045)	0.368
Neck circumference	–	1.099 (1.061–1.138)	< 0.001	1.017 (0.953–1.085)	0.607
Age/10	–	1.901 (1.707–2.118)	< 0.001	2.136 (1.834–2.487)	< 0.001
Drinking	No	Ref	Ref	Ref	Ref
	Yes	0.995 (0.780–1.268)	0.965	–	–
Smoking	No	Ref	Ref	Ref	Ref
	Yes	1.222 (0.963–1.551)	0.099	–	–
High-salt diet	No	Ref	Ref	Ref	Ref
	Yes	1.337 (1.025–1.746)	< 0.05	1.083 (0.776–1.511)	0.639
High-fat diet	No	Ref	Ref	Ref	Ref
	Yes	1.277 (0.99–1.646)	0.060	–	–
Poor sleep quality	No	Ref	Ref	Ref	Ref
	Yes	1.348 (1.063–1.710)	< 0.05	1.058 (0.784–1.428)	0.711
Sedentariness	No	Ref	Ref	Ref	Ref
	Yes	0.842 (0.661–1.072)	0.163	–	–
Emotionally stable	No	1.217 (0.939–1.577)	0.139	–	–
	Yes	Ref	Ref	Ref	Ref
Mental stress	No	Ref	Ref	Ref	Ref
	Yes	1.257 (0.968–1.633)	0.086	–	–
Smoking amount	–	1.383 (1.109–1.723)	< 0.01	1.152 (0.869–1.527)	0.325
Memory decline	No	Ref	Ref	Ref	Ref
	Yes	1.430 (1.109–1.844)	< 0.01	1.070 (0.777–1.474)	0.679
Inattention	No	Ref	Ref	Ref	Ref
	Yes	1.048 (0.826–1.331)	0.698	–	–
Epworth sleepiness scale	–	1.070 (1.048–1.093)	< 0.001	1.021 (0.993–1.049)	0.145
Course of snoring	–	1.068 (1.051–1.085)	< 0.001	1.009 (0.986–1.033)	0.443
Course of choking	–	1.064 (1.041–1.088)	< 0.001	1.004 (0.971–1.037)	0.825
AHI	–	1.014 (1.009–1.018)	< 0.001	0.994 (0.982–1.006)	0.341
OAI	–	1.014 (1.010–1.019)	< 0.001	1.007 (0.995–1.019)	0.282
Minimum SaO_2_/10	–	0.638 (0.584–0.697)	< 0.001	0.552 (0.456–0.668)	< 0.001
CT90/10	–	1.160 (1.104–1.219)	< 0.001	0.842 (0.751–0.944)	< 0.01

AHI, apnea–hypopnea index; OAI, obstructive apnea index; SaO_2_, arterial oxygen saturation; CT90/10, percentage of time of SaO_2_ < 90%/10; OR, odds ratio; 95% CI, 95% credible interval.

### Performance of the machine learning algorithm

**FIGURE 2:**
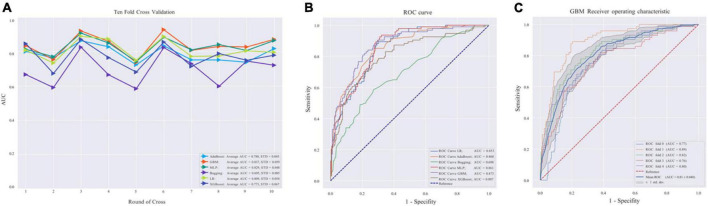
**(A)** Area under the curve (AUC) values of 10-fold cross-validation. **(B)** Validation of machine learning algorithms. **(C)** Receiver operating characteristic curve in the gradient boosting machine (GBM) model. AdaBoost, adaptive boosting; LR, logistic regression; Bagging, bootstrapped aggregating; MLP, multilayer perceptron; GBM, gradient boosting machine; XGBoost, extreme gradient boost; AUC, average area under the curve; ROC, receiver operating characteristic. AUC is used as an indicator of performance, the GBM model achieved the best predictive performance, and the Bagging model had the lowest predictive performance.

**FIGURE 3:**
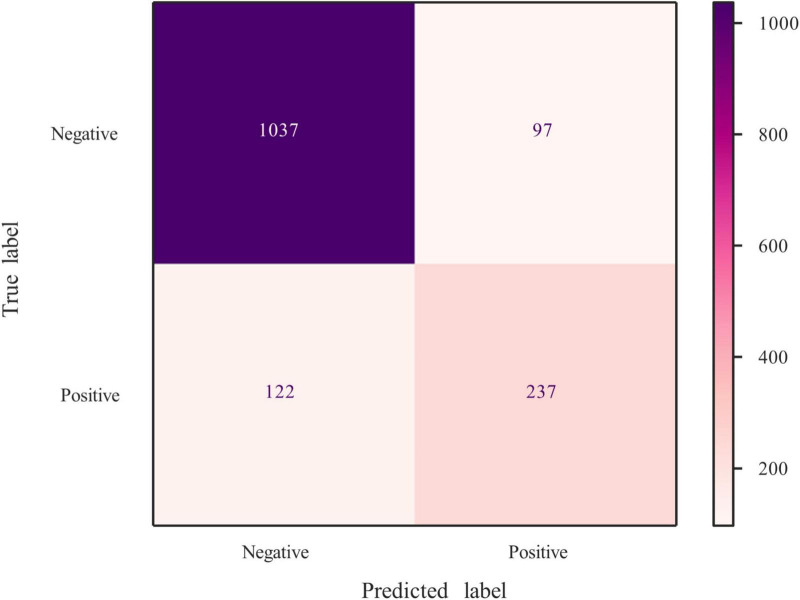
Confusion matrix of GBM. GBM, gradient boosting machine.

**FIGURE 4:**
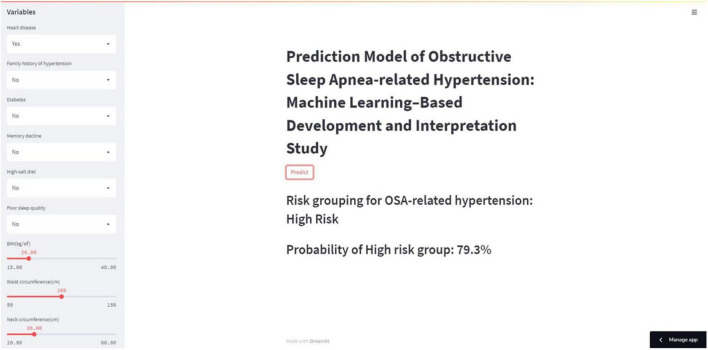
Web calculator for predicting OSA-related hypertension. OSA, obstructive sleep apnea.

The average AUC of the six models determined by 10-fold cross-validation is displayed in Figure 2A, with the GBM model achieving the best performance (AUC = 0.837). The model validation results based on the validation set are displayed in Figure 2B, and the GBM model still exhibited the best performance in predicting OSA-related hypertension (AUC = 0.873). Moreover, we further evaluated the stability and accuracy of GBM through five cross-validations, and the results reveal that the GBM has good stability (average AUC = 0.810 ± 0.048) (Figure 2C). The radar plot of the six ML models is shown in Supplementary Figure 1. A comparison of model performance on the validation set is shown in Table 3. Generally, all models performed satisfactorily in AUC, but not ideally in the sensitivity. Among them, the GBM exhibited the highest sensitivity at 0.713. Because GBM yielded the best results for AUC and sensitivity, we chose the GBM model as the final prediction model and then evaluated it (Figure 3). Meanwhile, on the basis of this model, we developed a prediction tool for the web, which can be accessed to further facilitate clinical use through an online risk calculator at https://shimunana-true-ml-vmz425.streamlitapp.com/ (Figure 4). The receiver operating characteristic properties of other ML models are shown in Supplementary Figure 2.

**TABLE 3:** Performance comparison of six machine learning (ML) models.

Model	F1 score	AUC	Accuracy	Sensitivity	Specificity
AdaBoost	0.757	0.860	0.832	0.553	0.925
LR	0.716	0.853	0.810	0.468	0.925
Bagging	0.481	0.698	0.759	0.353	0.916
MLP	0.719	0.861	0.807	0.489	0.914
GBM	0.841	0.873	0.885	0.713	0.943
XGBoost	0.719	0.807	0.807	0.489	0.914

AUC, area under the curve; AdaBoost, adaptive boosting; LR, logistic regression; Bagging, bootstrapped aggregating; MLP, multilayer perceptron; GBM, gradient boosting machine; XGBoost, extreme gradient boost; ML, machine learning.

### Model interpretability

**FIGURE 5:**
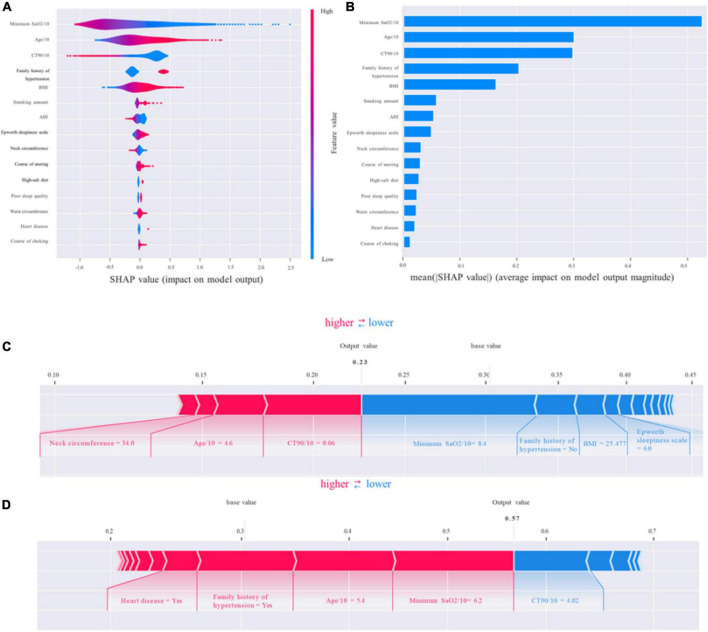
Shapley additive explanations (SHAP). **(A,B)** The standard and classified bar charts of the SHAP summary plots showed the influence of each parameter on the gradient boosting machine (GBM) model. **(C,D)** SHAP model explanation of two typical predictions. The features are ranked according to the sum of the SHAP values for all patients, and the SHAP values are used to show the distribution of the effect of each feature on the GBM model outputs. Each dot represents a case in the dataset. The color of a dot indicates the value of the feature, with blue indicating the lowest range and red the highest range. The horizontal axis shows the corresponding SHAP value of the feature. A positive SHAP value contributes to the prediction of rupture and vice versa. SHAP, Shapley additive explanations; GBM, gradient boosting machine; SaO_2_, arterial oxygen saturation; BMI, body mass index; AHI, apnea–hypopnea index; CT90/10, percentage of time of SaO_2_ < 90%/10.

To identify the features that influenced the prediction model the most, we illustrated the SHAP summary plot of GBM and the top 15 features of the prediction model in decreasing order (Figures 5A,B). The SHAP summary plot shows that age/10, family history of hypertension, minimum SaO_2_/10, BMI, and CT90/10 were the five most critical predictive features of the GBM model and had the most significant impact on the prediction results.

Shapley additive explanations (SHAP) values not only could show the contribution of each feature to the final prediction but also could effectively clarify and explain model predictions for individual patients. We provided two living examples to illustrate the role of the SHAP method in describing the machine learning model: a 46-year-old female patient who was diagnosed with OSA but with normal blood pressure and a 54-year-old male patient who was diagnosed with OSA-related hypertension (Figures 5C,D). The constructed model predicted the probability of OSA-related hypertension to be 23% and 57%, respectively. The model predicted the outcome as non-OSA-related hypertension for the female patient, which was consistent with the actual outcome (true negative). In addition, the model prediction result was OSA-related hypertension for the male patient, which was consistent with the actual situation (true positive).

## Discussion

The present study is the first study to assess the predictive performance of several machine learning algorithms for OSA-related hypertension, obtain a GBM model that can be used to predict OSA-related hypertension clinically, and explain the model. GBM is a commonly used ML algorithm with satisfactory performance in managing large and complex non-linear datasets and avoiding overfitting (27). Subsequently, we designed a network risk calculator based on the GBM model to estimate the probability of hypertension in individuals with OSA so as to help clinicians make targeted diagnoses and treatment plans, making precision medicine possible.

As hypothesized, our multivariate logistic regression suggested that BMI, age/10, and minimum SaO_2_/10 were significant independent risk factors for OSA-related hypertension, which converges with previous research. Pan et al. found that among police officers in southern China, the prevalence of OSA-related hypertension was associated with the age of the patients. However, their study population was small and occupation-specific (28). Furthermore, Natsios and colleagues reported that age, BMI, comorbidity, daytime SaO_2_, and indices of hypoxia during sleep were estimated to be the most precise predictors of hypertension (29). Additionally, because of the differences in study design and study population, we found some different results from previous studies. Family history of hypertension and CT90/10 were also found to be risk factors for OSA-related hypertension in our study. Interestingly, to further confirm how input factors contribute to the model, we calculated SHAP feature importance and feature effects. The importance of variables also showed that the BMI, age/10, and minimum SaO_2_/10, family history of hypertension, and CT90/10 were the most important input parameters that contribute to the predicted risk of OSA-related hypertension. This strongly demonstrates that these five variables were significant contributors to OSA-related hypertension, and proved the accuracy and reliability of the GBM model. Surely, a prospective study and animal experiments are essential to confirm the accuracy and reliability of our proposed model.

Interestingly, in addition to identifying several known risk factors, multivariate logistic regression and SHAP analysis also found that CT90/10, a variable that had been overlooked in previous cardiovascular studies, also plays an important role in OSA-related hypertension. Previous studies have shown a significant association between CT90 and Coronary Artery Calcium, cerebral small vessel disease and diabetic nephropathy (30–32), but the relationship between CT90 and hypertension has not been explored. The underlying causes by OSA and hypertension have not been fully elucidated. A few pathophysiological mechanisms have been suggested to participate in it, such as elevated sympathetic nervous system activity, renin-angiotensin aldosterone system activity, endothelial dysfunction, inflammation, and metabolic dysregulation (33). And López-Cano et al. showed a positive and significant association between the nocturnal concentration of urine metanephrines and the CT90 (34), suggesting that CT90 may influence sympathetic activity. And this also explains the important role of CT90 in OSA-related hypertension, and needs more attention in the future. Surprisingly, in our statistical model, AHI, as a diagnostic indicator of adult OSA, participates weakly. Whether there is a dose–response relationship between the severity of OSA and the cumulative incidence of hypertension has been debated. The Wisconsin Sleep Cohort Study discovered a dose–response association between OSA and the presence of hypertension 4 years later (35). At the same time, the Sleep Heart Health Study and the Victoria Sleep Cohort Study found that the relationship between hypertension and OSA was no longer significant after age and BMI were controlled for O’Connor et al. (36) and Cano-Pumarega et al. (37). Additionally, AHI is a simple measure of the average number of respiratory events (apneas and hypopneas) per hour of sleep, and it does not reflect adequately the various phenotypes and comorbidities of OSA. Our results disclosed that blood oxygen indicators (e.g., minimum SaO_2_/10 and CT90/10) might be better predictors of OSA-related hypertension than AHI.

Notably, the risk for OSA-related hypertension is increased most by family history of hypertension in the multivariate logistic regression, followed by age/10. However, the SHAP analysis showed that minimum SaO_2_/10 has the highest predictive value for OSA-related hypertension. The discrepancy between multivariate logistic regression and SHAP values can be explained by the prevalence of a variable. Odds ratios were calculated only for patients associated with this variable, but the mean SHAP value for all patients was calculated. In addition, the average SHAP value was further used to evaluate the importance of features and rank them. Hence, variables with low impact and high prevalence will have low odds ratios but high SHAP values.

In our study, full integration of the standard clinical variables with Polysomnography parameters was performed during the construction of the ML model. The model can thus predict OSA-related hypertension risk stratification for the patient, using all relevant covariates rather than individual measures. Our approach was also validated with repeated 10-fold cross-validation to provide a robust estimation of prediction accuracy with minimal bias. The six models performed well, with AUC ranging from 0.698 to 0.873 and sensitivity from 0.353 to 0.713 in the test dataset. And the GBM prediction model with the highest AUC, accuracy, and sensitivity was identified as the final model for this study and clinical use. The GBM model with 0.873 AUC and 0.713 sensitivity proves good discrimination and stability. What’s more, we introduce the Shapley value to explain the GBM model. SHAP is a model-independent interpretation technique that interprets black box models globally and locally, and can provide a rational explanation for the prediction, which can significantly enhance the confidence of clinicians in the model.

However, despite our best efforts to improve it, this study still has some limitations. First, this is a single-center retrospective study, and the performance of machine learning algorithms may vary for datasets with different distributions of patient characteristics and various institutions. Therefore, more patients from multiple sources are required to validate our model’s robustness and repeatability in the future. Second, the undesirable sensitivity may be that the ML algorithm learns from input features, and some discreet relationships may have been lost because of unknown or disregarded features not registered by doctors. In the future, we will conduct prospective validation based on this model, continue to explore crucial risk factors for OSA-related hypertension, and modify the model further to improve the accuracy and reliability of the GBM prediction model.

## Conclusion

We established a risk prediction model for OSA-related hypertension patients using the ML method and demonstrated that the GBM model performs best among the six ML models. This prediction model could help to identify high-risk OSA-related hypertension patients, provide early and individualized diagnoses and treatment plans, protect patients from the serious consequences of OSA-related hypertension, and reduce the burden on society.

## Data availability statement

The raw data supporting the conclusions of this article will be made available by the authors, without undue reservation.

## Ethics statement

The studies involving human participants were reviewed and approved by the Ethics Committee of The Second Affiliated Hospital of Xi’an Jiaotong University (approval no. 2021031). Written informed consent for participation was not required for this study in accordance with the national legislation and the institutional requirements.

## Author contributions

YS, SW, and XR designed the research. XC and LM wrote the manuscript. YF, YY, HL, and LY collected the data. YX, ZC, and CZ performed data curation. YZ, LM, and WL analyzed and processed the data. XR directed the research and the guarantor of the manuscript and takes full responsibility for the integrity of the work, from its inception to the published manuscript. All authors reviewed the results and approved the final version of the manuscript.

## Abbreviations

OSA: obstructive sleep apnea
ML: machine learning
AUC: area under the curve
SHAP: Shapley additive explanations
SaO_2_: arterial oxygen saturation
OAI: obstructive apnea index
BMI: body mass index
GBM: gradient boosting machine
ABPM: ambulatory blood pressure monitoring
AHI: apnea–hypopnea index
CT90/10: percentage of time of SaO_2_ < 90%/10.
