# Supplementary material for: Prediction model of obstructive sleep apnea–related hypertension: Machine learning–based development and interpretation study
Source: Front Cardiovasc Med. 2022 Dec 5;9:1042996. doi: 10.3389/fcvm.2022.1042996 (PMC9760810; doi:10.3389/fcvm.2022.1042996)
Supplement: Supplementary file 1 [file Presentation_1.zip › supplementary material/Supplementary Material.pdf]

Prediction Model of Obstructive Sleep Apnea–related Hypertension: Machine Learning–Based Development and Interpretation Study

Methods

Study design and subjects

After the completion of polysomnography or home sleep apnea testing, patients with sleep duration greater than 6 hours were included, and take medications including anxiolytics, hypnotics, antidepressants, sedating H1 antihistamines, respiratory stimulants, diet aids, non-steroidal anti-inflammatory drugs, hormonal drugs or narcotic analgesics were excluded.

The definition of hypertension:

Hypertension was defined as a mean (average of the three measurements of the second office blood pressure reading) clinic SBP ≥ 140 mmHg and/or a mean clinic DBP ≥ 90 mmHg. Only patients with high blood pressure levels for the two consecutive days were defined as hypertensives in clinic. Ambulatory blood pressure testing is used to diagnose patients with OSA who have no history of hypertension but have elevated blood pressure at night. Ambulatory blood pressure testing define hypertension as a daytime SBP/DBP of 135/85 mm Hg, a nighttime SBP/DBP of 120/70 mm Hg, and a 24-hour SBP/DBP of 130/80 mm Hg(1).

Reference

1. Whelton PK, Carey RM, Aronow WS, Casey DE, Jr., Collins KJ, Dennison Himmelfarb C, et al. 2017 ACC/AHA/AAPA/ABC/ACPM/AGS/APhA/ASH/ASPC/NMA/PCNA Guideline for the Prevention, Detection, Evaluation, and Management of High Blood Pressure in Adults: A Report of the American College of Cardiology/American Heart Association Task Force on Clinical Practice Guidelines. Hypertension. 2018;71(6):e13-e115.

Tables

Table 1. Multivariate Logistic Regression Analyses

Variables	Category	B	SE	Wald	*P*	95%CI
**Heart disease**	No	Ref	Ref	Ref	Ref	Ref
	Yes	0.363	0.271	1.789	0.181	-0.168 - 0.894
**Family history of hypertension**	No	Ref	Ref	Ref	Ref	Ref
	Yes	1.684	0.155	117.883	<0.001	1.380 - 1.988
**Diabetes**	No	Ref	Ref	Ref	Ref	Ref
	Yes	0.615	0.349	3.108	0.078	-0.069 - 1.299
**Body mass index**	-	0.114	0.039	8.415	<0.01	0.038 - 0.190
**Waist circumference**	-	0.014	0.015	0.809	0.368	-0.015 - 0.043
**Neck circumference**	-	0.017	0.033	0.264	0.607	-0.048 - 0.082
**Age/10**	-	0.759	0.078	95.609	<0.001	0.606 - 0.912
**High-salt diet**	No	Ref	Ref	Ref	Ref	Ref
	Yes	0.080	0.170	0.220	0.639	-0.253 - 0.413
**Poor sleep quality**	No	Ref	Ref	Ref	Ref	Ref
	Yes	0.057	0.153	0.138	0.711	-0.243 - 0.357
**Smoking amount**	-	0.141	0.144	0.968	0.325	-0.141 - 0.423
**Memory decline**	No	Ref	Ref	Ref	Ref	Ref
	Yes	0.068	0.163	0.171	0.679	-0.251 - 0.387
**Epworth sleepiness scale**	-	0.021	0.014	2.122	0.145	-0.006 - 0.048
**Course of snoring**	-	0.009	0.012	0.589	0.443	-0.015 - 0.033
**Course of choking**	-	0.004	0.017	0.049	0.825	-0.029 - 0.037
**AHI**	-	-0.006	0.006	0.906	0.341	-0.018 - 0.006
**OAI**	-	0.007	0.006	1.159	0.282	-0.005 - 0.019
**Minimum SaO_2/10**	-	-0.594	0.097	37.507	<0.001	-0.784 - -0.404
**CT90/10**	-	-0.172	0.058	8.664	<0.01	-0.286 - -0.058
**Constant**	-	-6.167	1.408	19.192	<0.001	-

Note. AHI, apnea–hypopnea index; OAI, obstructive apnea index; SaO_2, arterial oxygen saturation; CT90/10, percentage of time of SaO_2 <90%/10; B: regression coefficient; SE, standard error; OR: odds ratio; 95%CI, 95% credible interval.

Figures

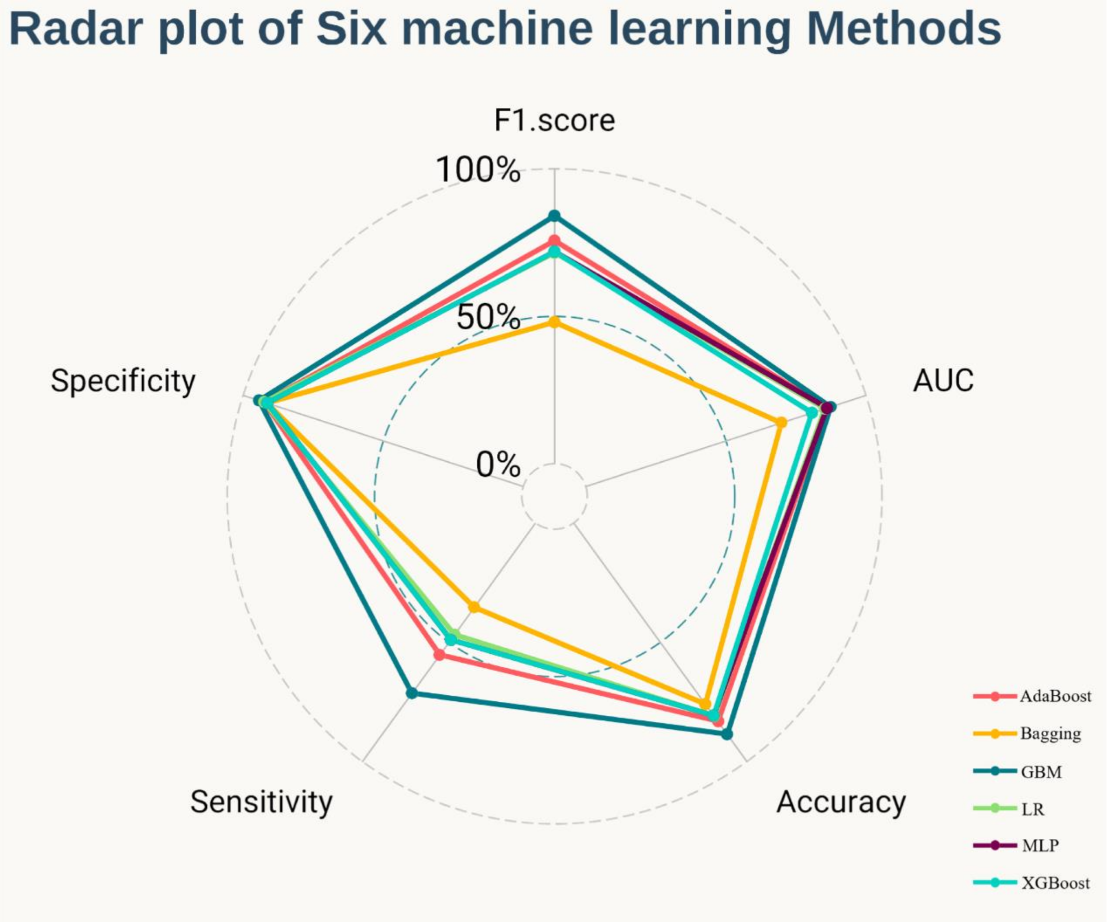

Figure S1. **Rader plot of six machine learning methods.** AdaBoost, adaptive boosting; LR, logistic regression; Bagging, bootstrapped aggregating; MLP, multilayer perceptron; GBM, gradient boosting machine; XGBoost, extreme gradient boost; AUC, average area under the curve.

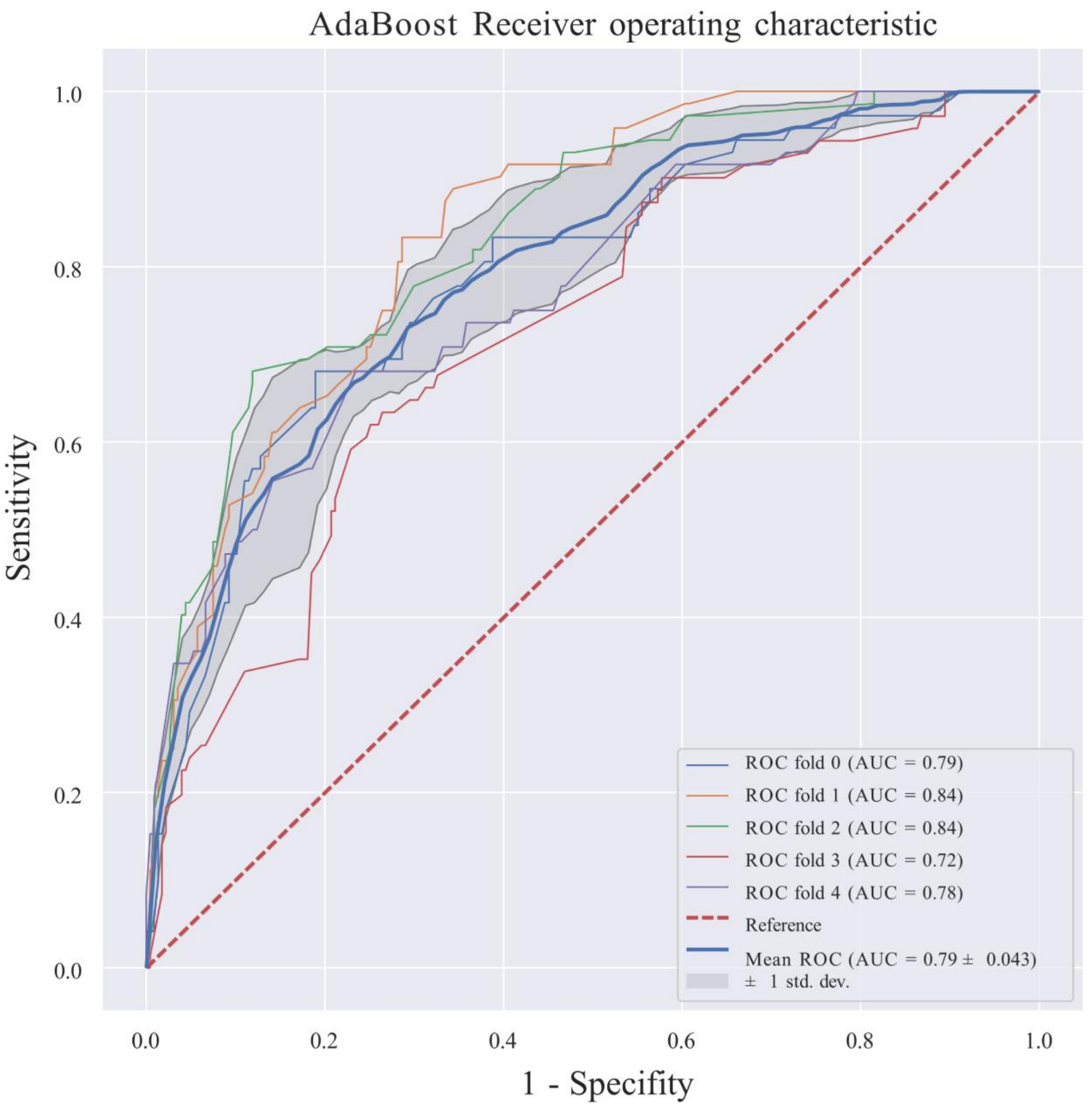

Figure S2. **AdaBoost receiver operating characteristic.** AdaBoost, adaptive boosting; AUC, average area under the curve; ROC, receiver operating characteristic.

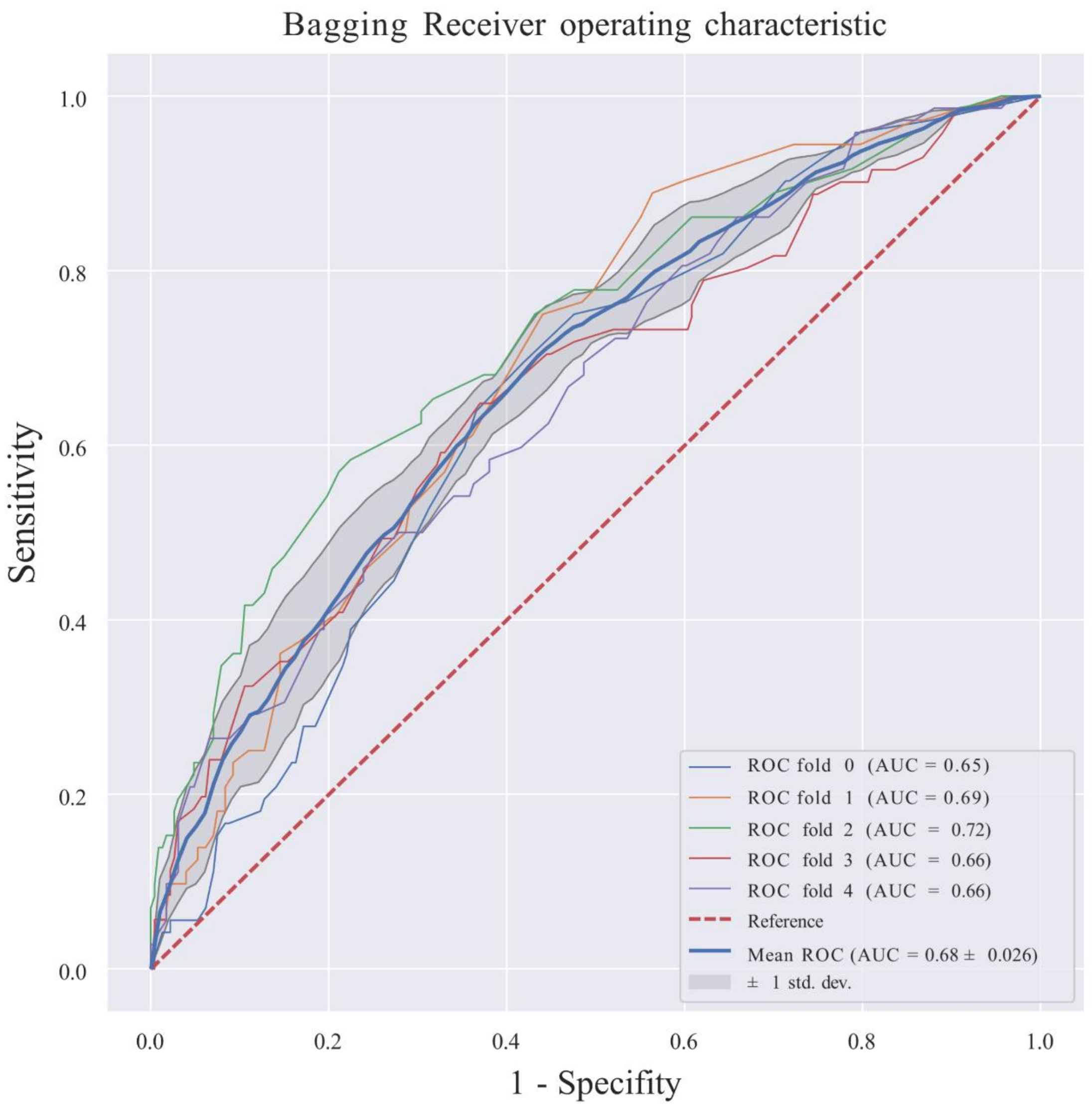

Figure S2. **Bagging receiver operating characteristic.** Bagging, bootstrapped aggregating; AUC, average area under the curve; ROC, receiver operating characteristic.

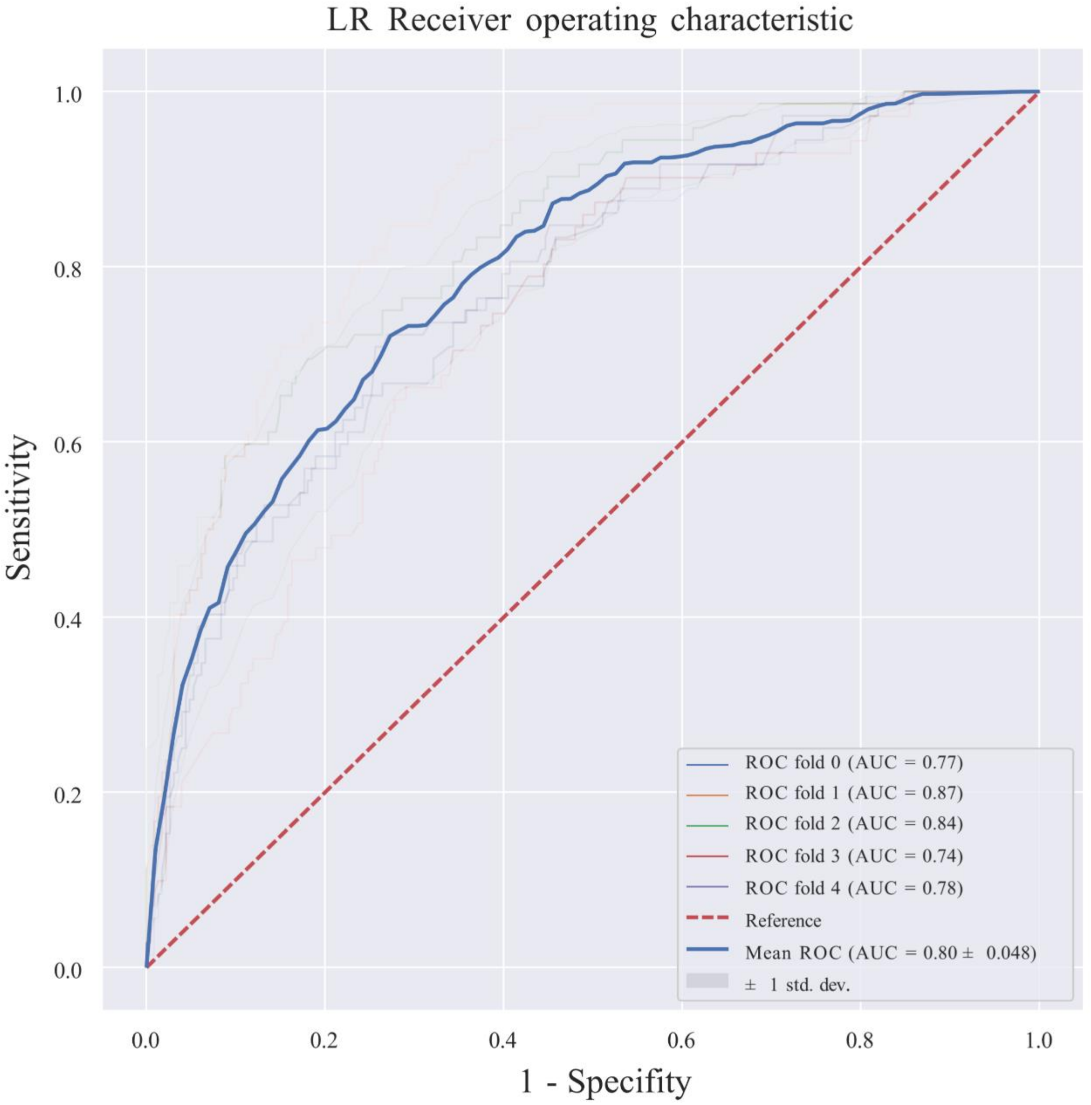

Figure S2. **LR receiver operating characteristic.** LR, logistic regression; AUC, average area under the curve; ROC, receiver operating characteristic.

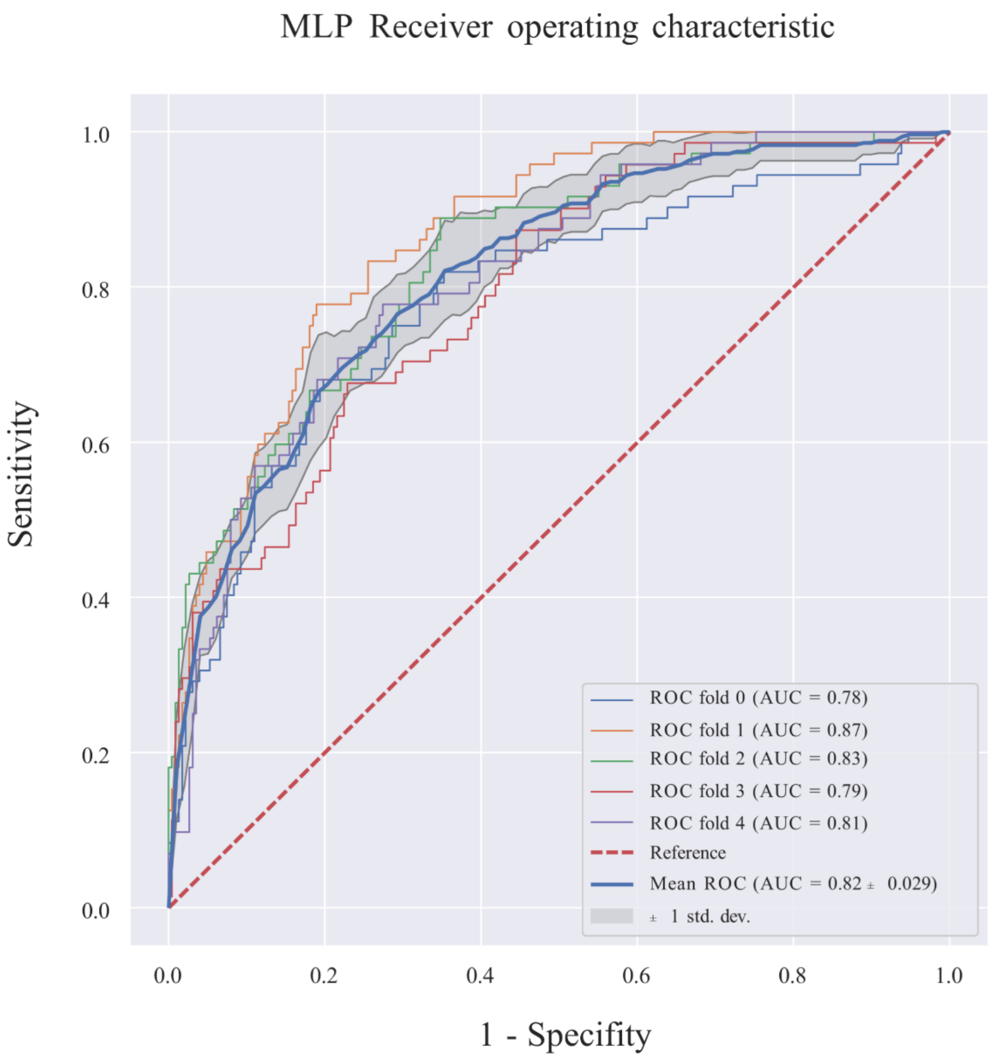

Figure S2. **MLP receiver operating characteristic.** MLP, multilayer perceptron; AUC, average area under the curve; ROC, receiver operating characteristic.

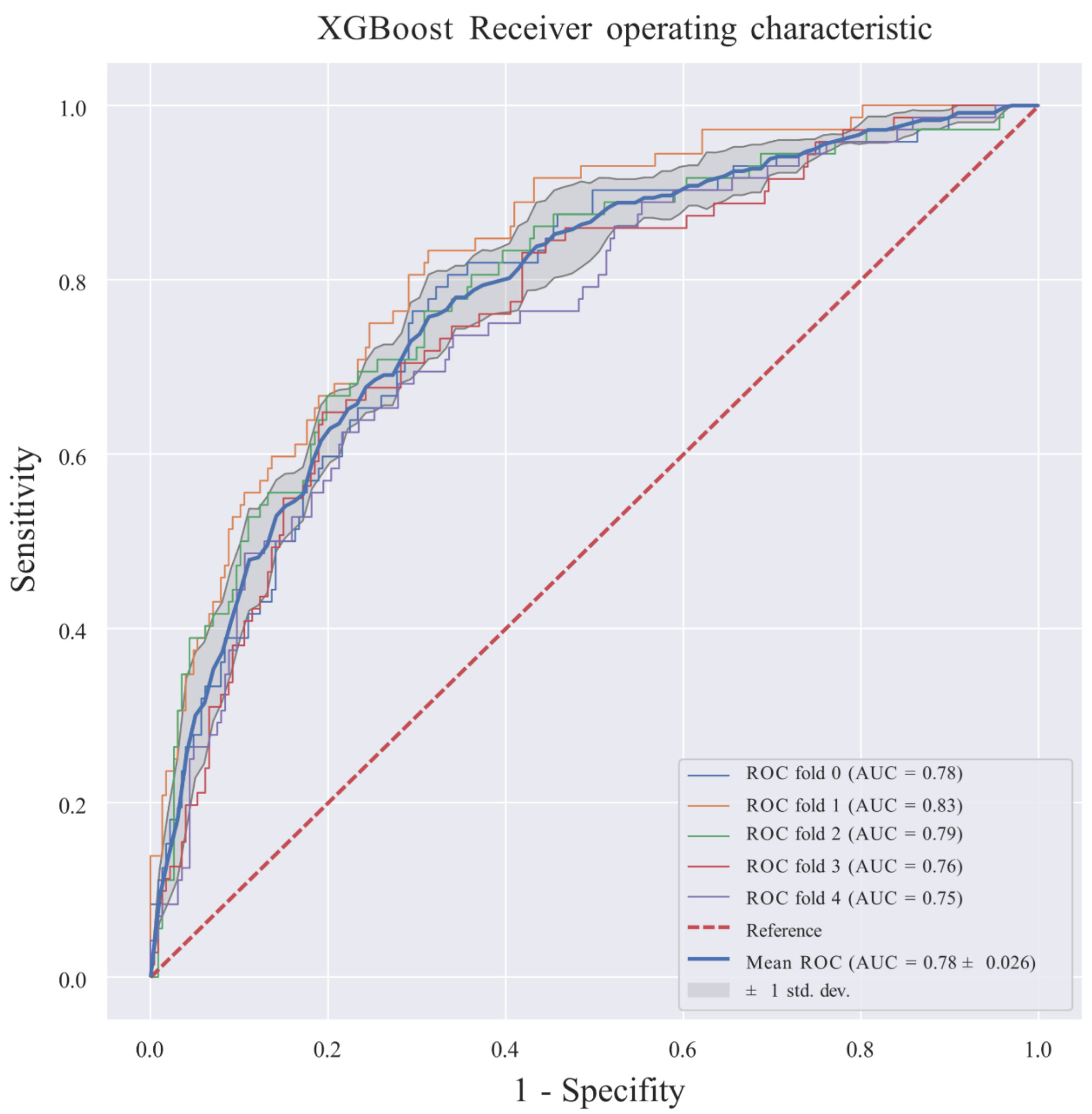

Figure S2. **XGBoost receiver operating characteristic.** XGBoost, extreme gradient boost; AUC, average area under the curve; ROC, receiver operating characteristic.
