# Supplementary figures and images for: Prediction model of obstructive sleep apnea–related hypertension: Machine learning–based development and interpretation study
Source: Front Cardiovasc Med. 2022 Dec 5;9:1042996. doi: 10.3389/fcvm.2022.1042996 (PMC9760810; doi:10.3389/fcvm.2022.1042996)

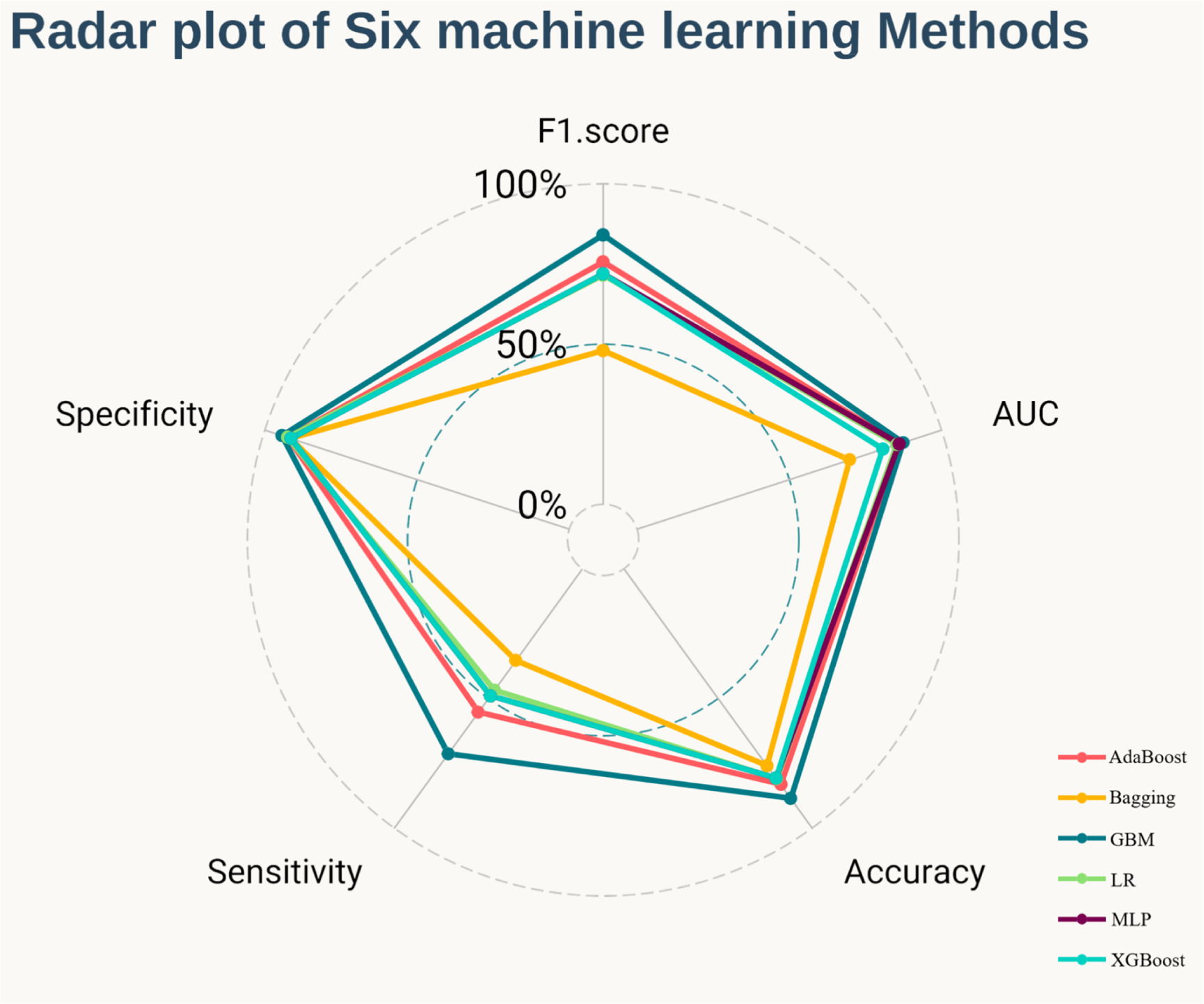

Supplement: Supplementary file 1 [file Presentation_1.zip › supplementary material/supplementary material. Figure 1.tif]

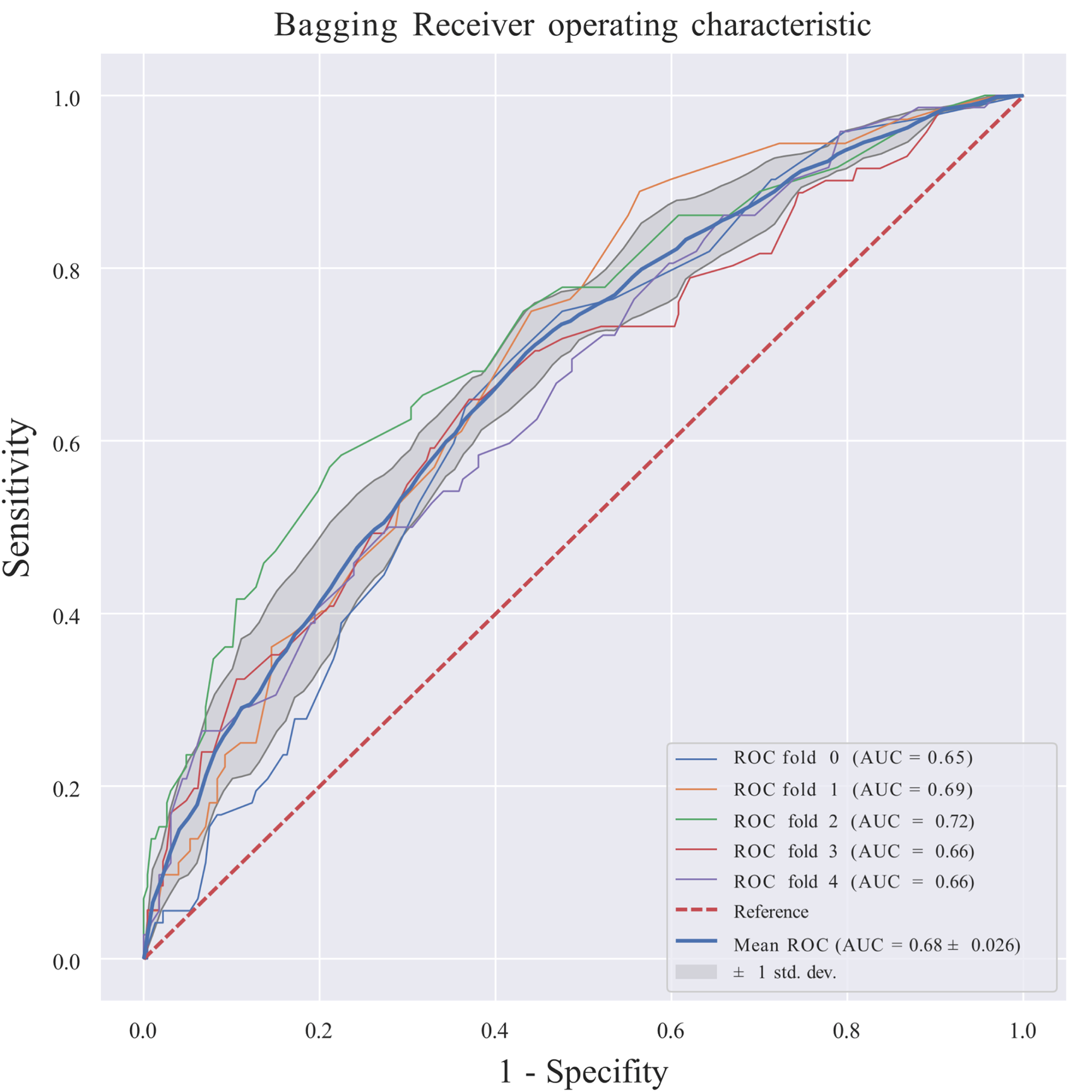

Supplement: Supplementary file 1 [file Presentation_1.zip › supplementary material/supplementary material; Figure_XGBoost.tif]

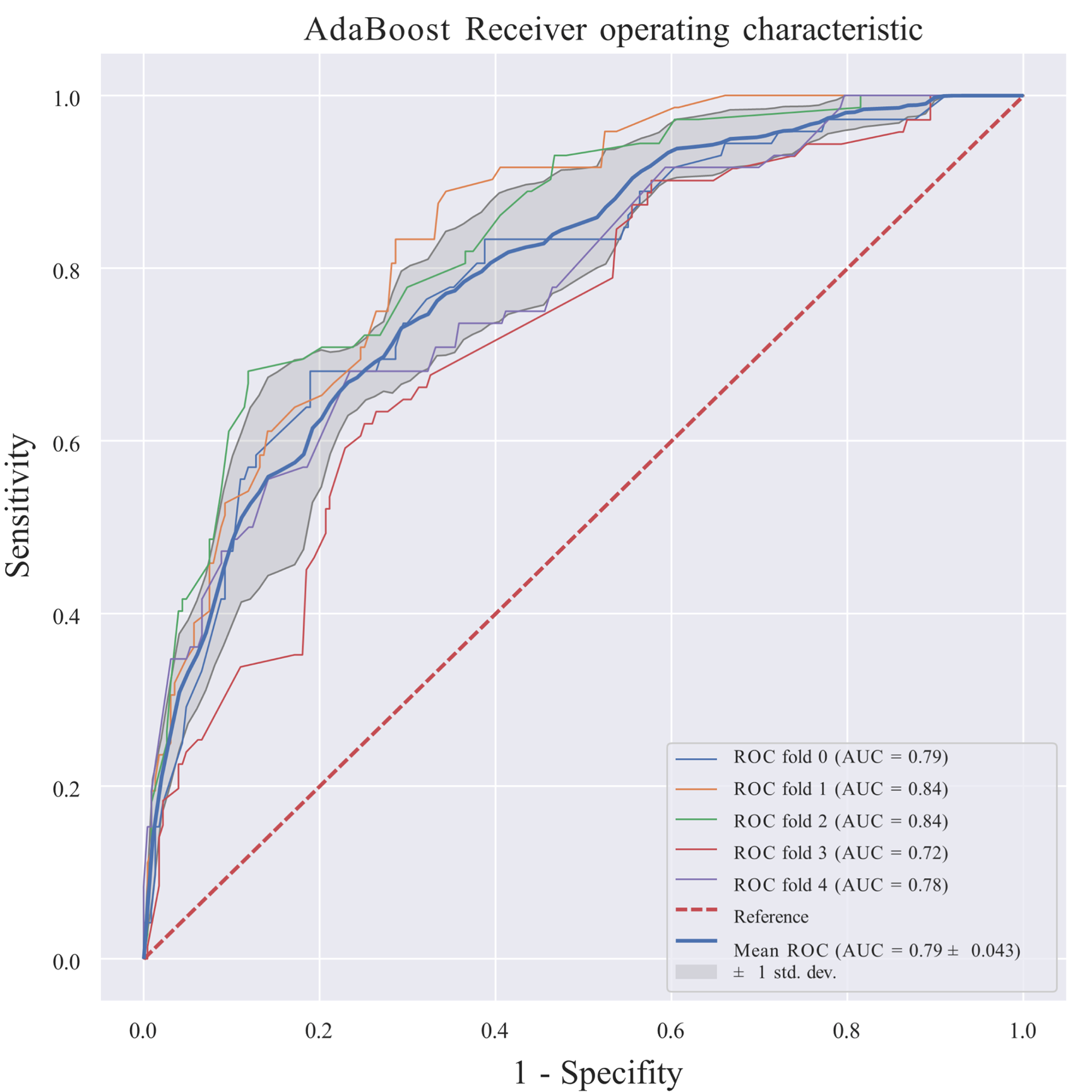

Supplement: Supplementary file 1 [file Presentation_1.zip › supplementary material/supplementary material: Figure_AdaBoost.tif]

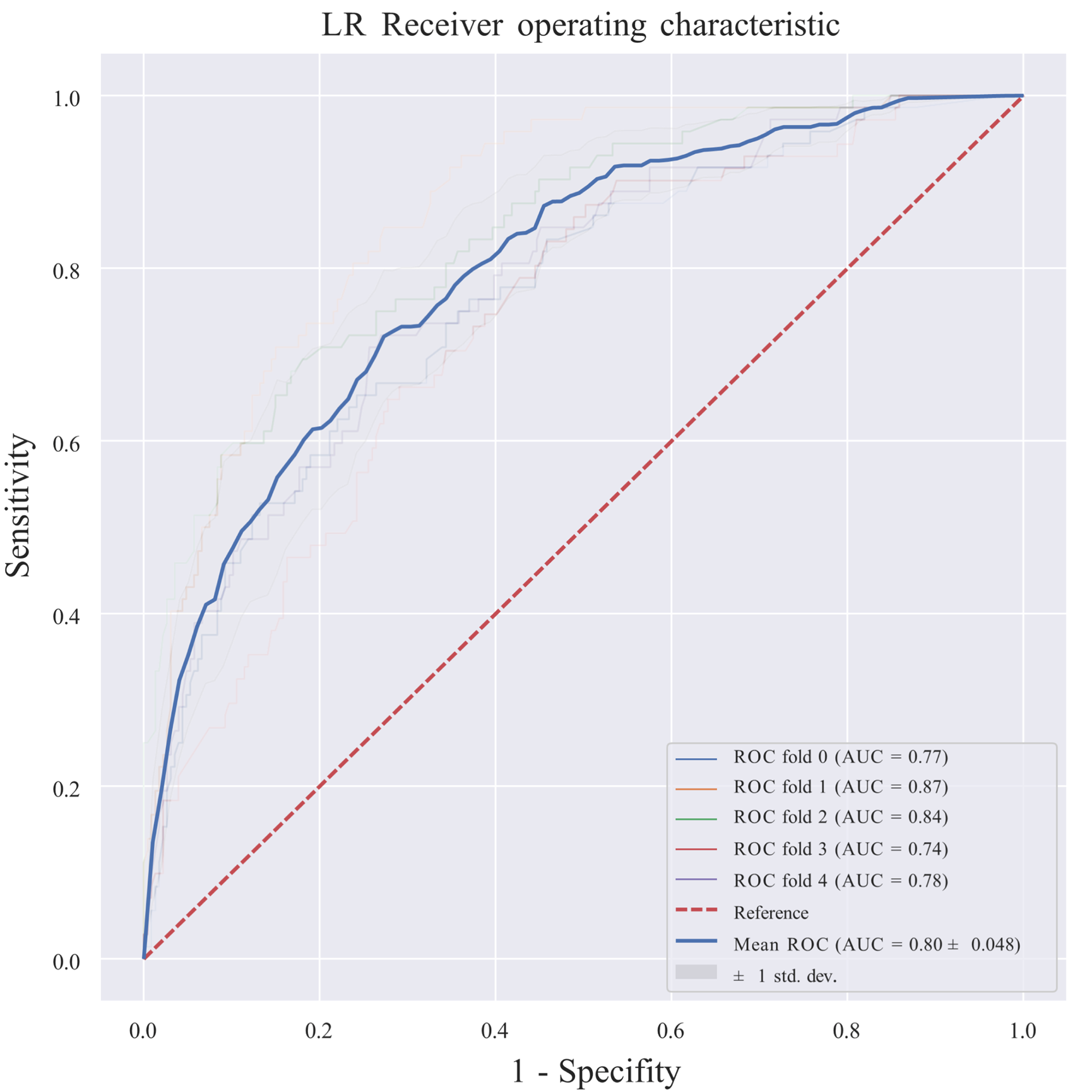

Supplement: Supplementary file 1 [file Presentation_1.zip › supplementary material/supplementary material: Figure_LR.tif]

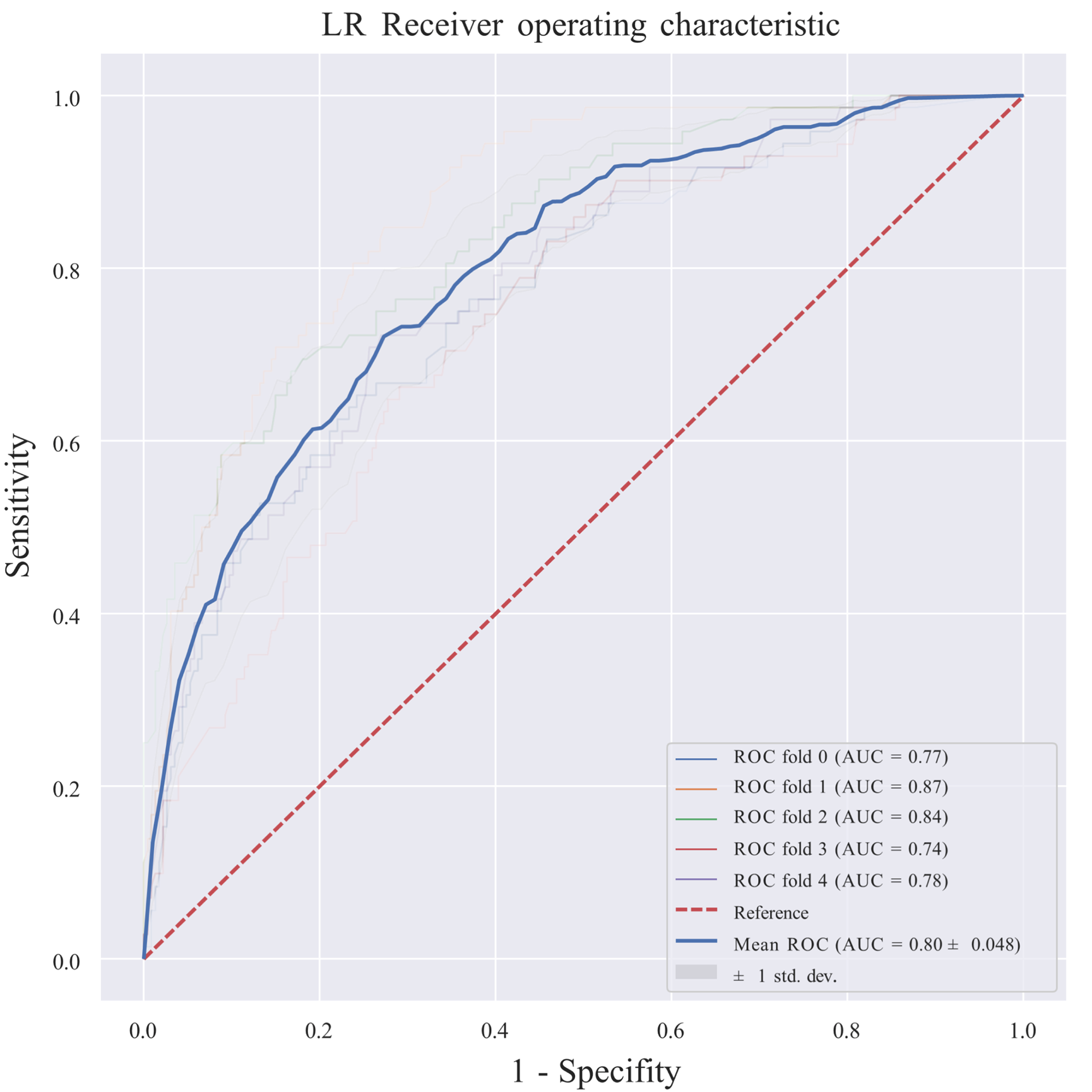

Supplement: Supplementary file 1 [file Presentation_1.zip › supplementary material/supplementary material:Figure MLP .tif]

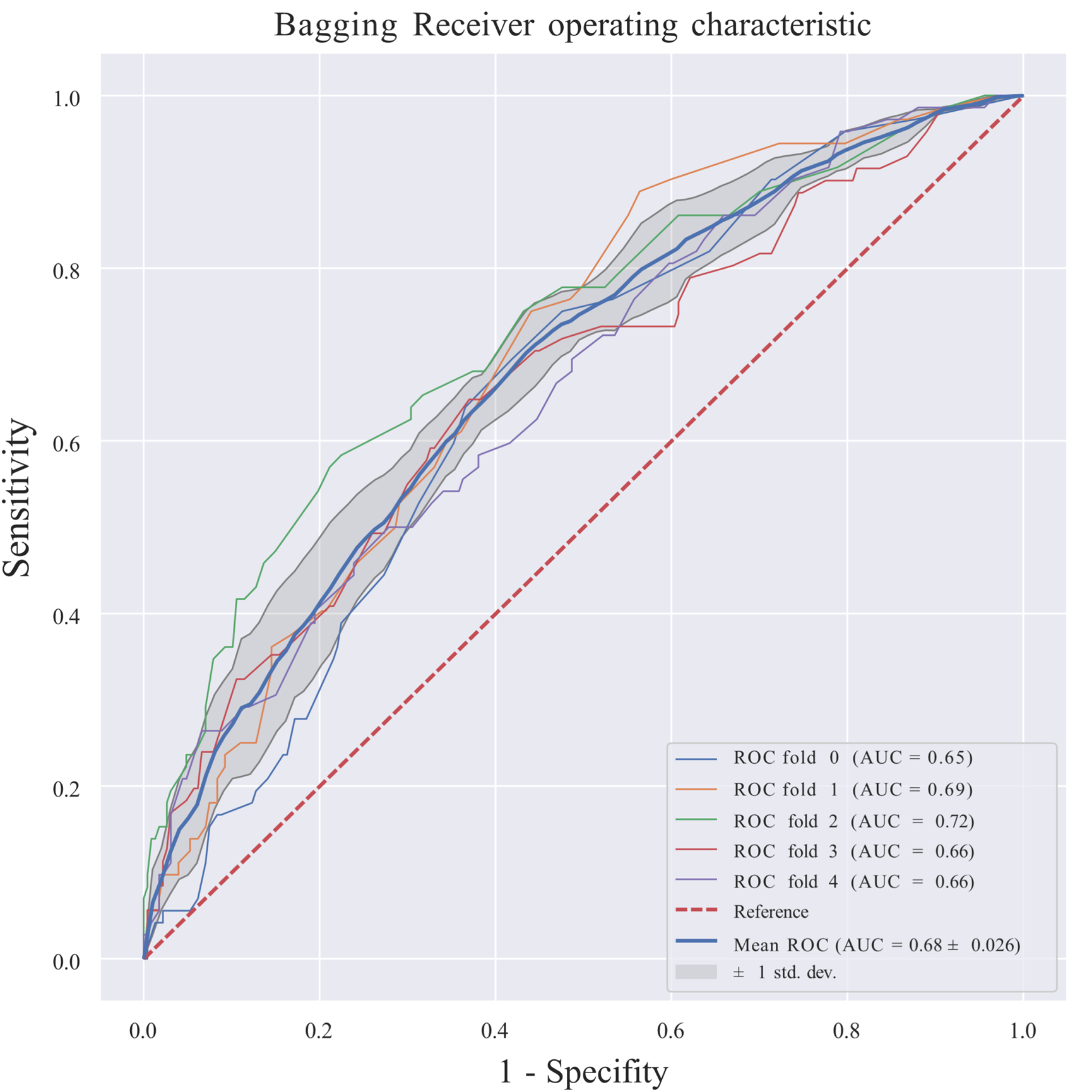

Supplement: Supplementary file 1 [file Presentation_1.zip › supplementary material/supplementary material:Figure_Bagging.tif]
